# Complete mitochondrial genome sequence of Chenghua pig (*Sus Scrofa*) and its phylogenetic analysis

**DOI:** 10.1080/23802359.2016.1197058

**Published:** 2016-12-09

**Authors:** Lin-Yuan Shen, Shun-Hua Zhang, Li Zhu

**Affiliations:** College of Animal Science and Technology, Sichuan Agricultural University, Chengdu, Sichuan, China

**Keywords:** Chenghua pig, genome, mitochondria

## Abstract

Chenghua pig is one of the famous indigenous breeds of Sichuan province in China. In the study, the complete mitochondrial genome of Chenghua pig was first determined. The total mitochondrial genome is 16,760 bp in length and its overall base composition of the circle genome is A (34.71%), T (25.79%), C (26.20%) and G (13.30%), respectively, indicating an A–T (60.50%)-rich feature. It contains the typical structure, including 22 transfer RNA genes, 13 protein-coding genes, 2 ribosomal RNA genes and a major non-coding control region (D-Loop region). The D-loop region contains two different repeat motifs. According to the phylogenetic tree, we can infer that it is reasonable to divide Chenghua pig into the genus *Sus Scrofa* in southwest China. The mitochondrial genome would play an important role in population genetics and phylogenetic analysis of *Sus Scrofa.*

Chenghua pig is one of the endangered indigenous pig breeds in Sichuan province in China. The population size is sharply shrink by reason of foreign pig breeds large-scale feeding in china. Here, we first report the mitochondrial genome of this species and its sequence was deposited in GenBank (accession number: KP765603). The specimen of Chenghua pig was collected from Qionglai Country, Sichuan Province, China (30.42°N, 103.47°E). The specimen (DNA and tissue) was deposited in the Museum of Sichuan Agricultural University (Voucher numbers MSAU-20151404).

The total length of Chenghua pig mitogenome is 16,760 bp. It is consist of 36 genes, including 22 transfer RNA genes, 13 protein-coding genes, 2 ribosomal RNA genes and a major noncoding control region (D-Loop region) (Table 1). The distribution and length of these genes is the same as that found in other pig breeds (Yang et al. 2014). The total base composition of Chenghua pig mitochondrial genome is A (34.71%), T (25.79%), C (26.20%) and G (13.30%), which is similar to other breeds (Shen et al. 2015). Furthermore, the whole mitogenome sequence has six overlaps and eleven spacers. The longest overlap is located between *ATP8* and *ATP6* with a length of 43bp, and the longest spacer is located between tRNA^Tyr^ and tRNA^Asn^ and its length is 32bp (Table 1).

**Table 1.** Organization of the mitochondrial genome of Chenghua pig.

	Position				Codon				
Gene	Start	End		Size	Start	Stop^a^	Anti-codon	Strand	Space/overlap+
Dloop	1	1324		1324	–	–	–	H	/
tRNA^Phe^	1325	1394		70	–	–	GAA	H	0
12s rRNA	1395	2355		961	–	–	–	H	0
tRNA^Val^	2356	2423		68	–	–	TAC	H	0
16s rRNA	2424	3993		1570	–	–	–	H	0
tRNA^Leu^	3994	4068		75	–	–	TAA	H	0
*ND1*	4071	5025		955	ATG	T––	–	H	2
tRNA^Ile^	5026	5094		69	–	–	GAT	H	0
tRNA^Gln^	5092	5164		73	–	–	TTG	L	−3^b^
tRNA^Met^	5166	5235		70	–	–	CAT	H	1
*ND2*	5236	6277		1042	ATT	T––	–	H	0
tRNA^Trp^	6278	6345		68	–	–	TCA	H	0
tRNA^Ala^	6352	6419		68	–	–	TGC	L	6
tRNA^Asn^	6421	6495		75	–	–	GTT	L	1
tRNA^Cys^	6528	6593		66	–	–	GCA	L	32
tRNA^Tyr^	6593	6658		66	–	–	GTA	L	−1^b^
*COX1*	6660	8204		1545	ATG	TAA	–	H	1
tRNA^Ser^	8208	8278		71	–	–	TGA	L	3
tRNA^Asp^	8284	8351		68	–	–	GTC	H	5
*COX2*	8352	9039		688	ATG	T––	–	H	0
tRNA^Lys^	9040	9106		67	–	–	TTT	H	0
*ATPase8*	9108	9311		204	ATG	TAA	–	H	1
*ATPase6*	9269	9949		681	ATG	TAA	–	H	−43^b^
*COX3*	9949	10,732		784	ATG	T–	–	H	−1^b^
tRNA^Gly^	10,733	10,801		69	–	–	TCC	H	0
*ND3*	10,802	11,147		346	ATA	T––	–	H	0
tRNA^Arg^	11,149	11,217		69	–	–	TCG	H	1
*ND4L*	11,218	11,514		297	GTG	TAA	–	H	0
*ND4*	11,508	12,885		1378	ATG	T––	–	H	−7^b^
tRNA^His^	12,886	12,954		69	–	–	GTG	H	0
tRNA^Ser^	12,955	13,013		59	–	–	GCT	H	0
tRNA^Leu^	13,014	13,083		70	–	–	TAG	H	0
*ND5*	13,084	14,904		1821	ATA	TAA	–	H	0
*ND6*	14,888	15,415		528	ATG	TAA	–	L	−17^b^
tRNA^Glu^	15,416	15,484		69	–	–	TTC	L	0
*Cytb*	15,489	16,628	1140		ATG	AGA		H	4
tRNA^Thr^	16,629	16,696		68	–	–	TGT	H	0
tRNA^Pro^	16,697	16,760		64	–	–	TGG	L	0

^a^T– means incomplete termination codon.

^b^Negative numbers indicate overlapping nucleotides.

To further confirm the new determined mitogenome sequences, we downloaded another 11 *Sus scrofa* mitochondrial DNA sequences from the GenBank (Figure 1). A neighbor-joining phylogenetic tree was constructed based on 13 protein-coding genes using the MEGA 6.06 (Tamura et al. 2013). On the NJ tree, Chenghua pig was closely related to domestic pigs in southwest China. This result was consistent with the geographical distribution of Chenghua pig.

**Figure 1.**
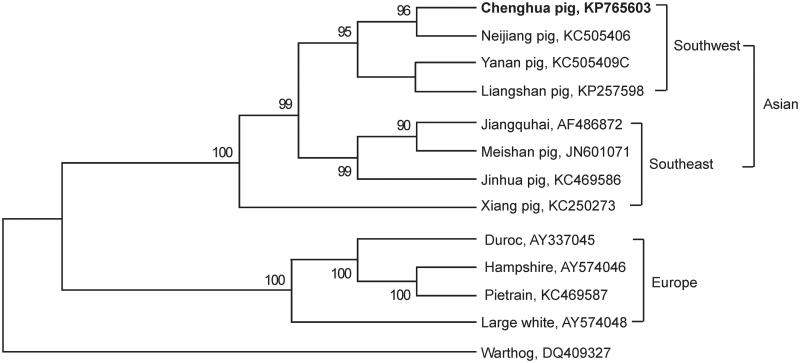
Neighbor-joining (NJ) tree based on combining 13 protein-coding gene sequences of 13 species by using MEGA 6.06 program. The NJ bootstrap for 10 000 replicates was indicated in each node. The Warthogs was chosen as the outgroup.
